# Kinase-Based Screening of Marine Natural Extracts Leads to the Identification of a Cytotoxic High Molecular Weight Metabolite from the Mediterranean Sponge *Crambe tailliezi*

**DOI:** 10.3390/md17100569

**Published:** 2019-10-09

**Authors:** Thi-Ngoc-Dung Nguyen, Omid Feizbakhsh, Estelle Sfecci, Blandine Baratte, Claire Delehouzé, Adrien Garcia, Corentin Moulin, Pierre Colas, Sandrine Ruchaud, Mohamed Mehiri, Stéphane Bach

**Affiliations:** 1 Integrative Biology of Marine Models Laboratory (LBI2M), Sorbonne Université, CNRS, UMR 8227, Station Biologique de Roscoff, CS 90074, 29688 Roscoff Cedex, France; dnguyenthingoc@sb-roscoff.fr (T.-N.-D.N.); omid.feizbakhsh@sb-roscoff.fr (O.F.); blandine.baratte@sb-roscoff.fr (B.B.); claire.delehouze@seabelife.com (C.D.); colas@sb-roscoff.fr (P.C.); 2 Department of Chemical Analysis and Drug Quality Control, Faculty of Pharmacy, University of Medicine and Pharmacy, Ho Chi Minh City 70000, Vietnam; 3 Marine Natural Products Team, Université Côte d’Azur, CNRS, Institut de Chimie de Nice, UMR 7272, 06108 Nice, France; estelle.sfecci@univ-cotedazur.fr (E.S.); adrien.garcia@univ-cotedazur.fr (A.G.); corentin.moulin@univ-cotedazur.fr (C.M.); 4 Sorbonne Université, CNRS, Station Biologique de Roscoff, FR 2424, Plateforme de criblage KISSf (Kinase Inhibitor Specialized Screening facility), Place Georges Teissier, 29682 Roscoff, France; 5 SeaBeLife Biotech, Place Georges Teissier, 29682 Roscoff, France

**Keywords:** Screening, kinase inhibition, regulated cell death, RIP1-independent cell death, marine natural products, *Crambe tailliezi*

## Abstract

Regulated cell death (RCD) results from the activation of one or more signal transduction modules both in physiological or pathological conditions. It is now established that RCD is involved in numerous human diseases, including cancer. As regulated cell death processes can be modulated by pharmacological tools, the research reported here aims to characterize new marine compounds acting as RCD modulators. Protein kinases (PKs) are key signaling actors in various RCDs notably through the control of either mitosis (e.g., the PKs Aurora A and B) or necroptosis (e.g., RIPK1 and RIPK3). From the primary screening of 27 various extracts of marine organisms collected in the Mediterranean Sea, an extract and subsequently a purified high molecular weight compound dubbed P3, were isolated from the marine sponge *Crambe tailliezi* and characterized as a selective inhibitor of PKs Aurora A and B. Furthermore, P3 was shown to induce apoptosis and to decrease proliferation and mitotic index of human osteosarcoma U-2 OS cells.

## 1. Introduction

“Live or die” is the binary choice that a cell has to make in order to maintain the balance in the functions of a multicellular organism (also known as the homeostasis). Thus, similarly to the control of cell division, cell death is also tightly regulated [1]. The concept of “programmed cell death (PCD)”, which first appeared in the early 1960s, designates the natural process of regulated cell death that occurs during organisms’ development and tissue homeostasis [2,3]. PCD is now considered as a particular form of “regulated cell death” (RCD) that occurs in strictly physiological scenarios [4]. Death signaling pathways can be activated by internal or external signals. It is a natural process to eliminate undesirable cells including tumor ones. Thus, RCD plays a crucial role in either physiological or pathological conditions. Abnormal RCD can cause disorders such as cancer, inflammatory diseases, and neurodegenerative diseases [1,5]. Apoptosis was the first RCD described (1972) [6] and the most studied until now. The classical apoptosis pathway is triggered and mediated by cysteine-aspartic proteases called caspases [7]. The main goal in cancer chemotherapy is to induce apoptosis but many cancer cells gain the ability to overcome RCD by mutations giving them a survival advantage. Therefore, there is an urgent need for new therapeutic strategies based on new potent anticancer molecules activating canonical and noncanonical cell-death subroutines [8]. Indeed, recently, several groups have discovered and described other types of RCD that can be targeted, like necroptosis, autophagy, ferroptosis, or death by mitotic catastrophe [4,9]. These alternative RCD pathways may represent opportunities to trigger cell death when facing apoptosis resistance [10]. However, various open questions need to be addressed regarding their controls and regulatory crosstalk. Therefore, the characterization of new chemical triggers of RCD can kill two birds with one stone: (i) deciphering the molecular mechanisms leading to cell death and (ii) characterizing new putative drugs for cancer therapy.

**Figure 1:**
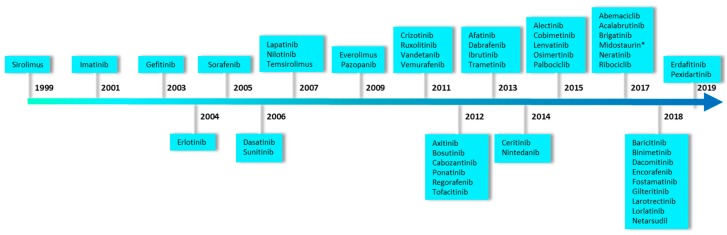
Food and Drug Administration (FDA)-approved protein kinase inhibitors as of August 2019. This timeline was performed using the data reported in Roskoski R., 2019. * Midostaurin is a derivative of a marine natural compound.

The great potential of marine products for human therapy was discovered in the 1950s with the discovery of two nucleosides spongothymidine and sponguridine from the Caribbean sponge *Cryptotethia crypta* [11,12,13]. Nowadays, it is well known that marine organisms can be new sources of drugs modulating various RCD processes [14]. Marine sponges (and their associated microbiota) are among the most promising group because of the chemical diversity of their secondary metabolites and their strong bioactivity as cytotoxic agents or protein kinase inhibitors (PKIs), among other activities [15,16,17]. Protein kinases are involved in the regulation of numerous cellular processes, often in response to an external stimulus. This family of enzymes has become one of the most important suppliers of drug targets and perhaps up to one third of drug discovery efforts worldwide are focused on the discovery of new PKIs [18]. The number of approved PKIs continues to grow and as of August 2019, 50 drugs have reached the US market (Figure 1), 85% of which are used for the treatment of malignancies [18]. More than 200 orally effective PKIs are currently in clinical trials worldwide (a complete and updated listing of PKIs in clinical trials can be found at www.icoa.fr/pkidb/) [19,20].

**Figure 2:**
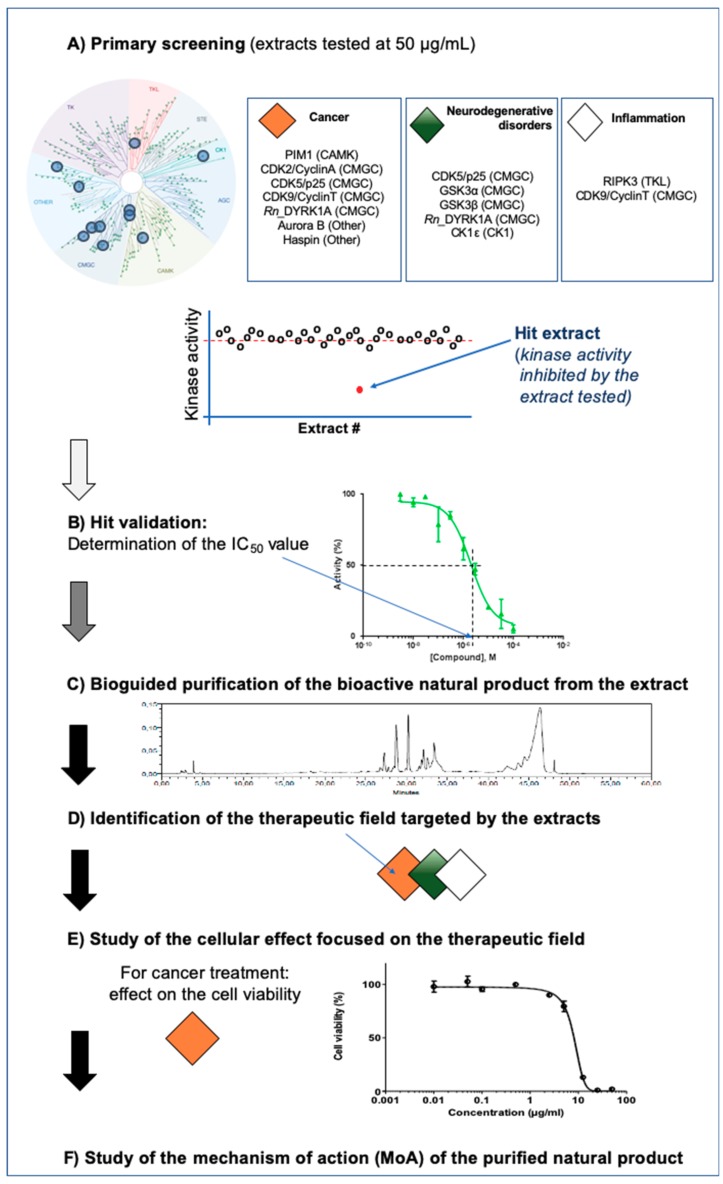
Workflow of the kinase-based screening assay of the marine extracts. (**A**) During the first step, a primary screening is performed against a disease-related panel of protein kinases. These targets are selected among the human kinome as mentioned on the figure by blue dots on the circular tree. This image was generated using TREEspot™ Software Tool (Eurofins DiscoverX Corporation, Fremont, CA, USA) and reprinted with permission from KINOMEscan^®^, a division of Eurofins DiscoverX Corporation (© DISCOVERX CORPORATION 2010). The codes reported on this figure indicate the subclasses of protein kinases: CMGC for CDKs, MAP kinases, GSK, and CDK-like kinases; AGC for protein Kinase A, C, and G families (PKA, PKC, PKG); CAMK for Ca2+/calmodulin-dependent protein kinases; CK1, Cell/Casein Kinase 1; STE, STE Kinases (Homologs of yeast STErile kinases); TKL, Tyrosine Kinases-Like; TK, Tyrosine Kinases. All protein kinases used here are human unless specified *Rn, Rattus norvegicus*. As mentioned on this theoretical scatter plot, an extract is selected as screening hit when it is shown to inhibit the kinase activity of the tested kinase. (**B**) In a second step, the IC_50_ values were then calculated from dose–response curves in order to validate the first hits. (**C**) Kinase inhibition was then used to guide the purification of the natural products from the crude extracts. From this step, the identification of the inhibited kinase drives the selection of both the therapeutic field (**D**) and the study of the cellular effects of the tested natural product (**E**). Here is shown the study of a compound that can inhibit kinase(s) involved in cancer (e.g., Aurora B kinase). The phenotype studied is the “*dose-dependent modulation of the cellular viability by the natural product*”. (**F**) In an optimal case, the final step is the study of the mechanism of action and, if possible, the characterization of the cellular targets of the selected marine products.

Manning et al. have first catalogued the 518+ protein kinases encoded by the human genome (the “kinome”). The kinome was classified based on protein sequences into eight typical groups (AGC, CAMK, CK1, CMGC, STE, TK, TKL, and other, see the legend of Figure 2 for details) and 13 atypical families [21].

In keeping with the high chemodiversity of PKIs described in marine sponges [15], a panel of 11 protein kinases was used as a filtering technique to associate a marine natural product with a disease-related cellular phenotype. Following this primary enzymatic screening campaign of an original collection of 27 extracts from marine organisms collected in the Mediterranean Sea, an extract from *Crambe tailliezi* and one of its purified product (P3) were shown to inhibit Aurora A and B kinases. This result oriented the analysis on the cellular phenotype induced by P3. The results obtained indicated that P3 could induce the programmed cell death of human cancer cell lines derived from leukemia and solid tumors affecting breast, colorectal, liver, bone, pancreas, and brain tissues. Moreover, the treatment of osteosarcoma U-2 OS cell line with P3 triggered apoptotic cell death.

## 2. Results

The methodological workflow used to characterize new bioactive extracts and purified fractions from a set of selected marine organisms is schematically represented in Figure 2. The primary screening was performed on a panel of defined targets (kinases), an approach often referred to as reverse chemical genetics/biology. Note here that the so-called forward chemical genetics/biology approaches entail phenotypic screening (e.g., used in neuropsychiatric drug discovery [22]).

### 2.1. Primary Screening of a Selected Set of Purified Marine Extracts Against a Panel of Disease-Related Kinases

The inhibitory activity of 27 extracts of marine organisms collected in the Mediterranean Sea (see the Materials and Methods section for details on the protocol used to obtain the extracts) was screened against a panel of 11 disease-related protein kinases (listed on Figure 2): Aurora B, CDK2/CyclinA, CDK5/p25, CDK9/CyclinT, CK1ε, GSK3α, GSK3β, Haspin, Pim1, RIPK3 kinases, and rat DYRK1A. The results obtained are reported in Table 1.

The screen revealed that several extracts from *Crambe tailliezi, Hexadella* sp., *Pseudaxinyssa* sp.*, Ircinia oros, Sarcotragus foetidus*, and *Sarcotragus spinosulus* showed interesting kinase inhibitory properties. The hit score obtained was very high: 50% calculated from the number of extracts inhibiting at least one kinase. Four ascidium extracts tested here (*Aplidium* sp., *Cystodytes* sp., *Halocynthia papillosa* and *Polysyncraton* sp.) did not show as much activity towards kinases as sponge extracts. As shown here, GSK3 isoforms α and β were the most frequently targeted kinases.

The extract E13 from *Crambe tailliezi* inhibited the mitotic kinase Aurora B whereas the extract E12 from *Crambe crambe*, which is also a member of the *Crambeidea* family, did not. In regard to the rather good selectivity toward the kinase tested, *Crambe tailliezi* was selected for further fractionation and isolation of the active natural products. Indeed, within the *Crambeidea* family, *Crambe tailliezi* [23], an encrusting marine sponge also distributed in the Mediterranean Sea, is only poorly studied for either its chemical composition or its biological effects, contrary to *Crambe crambe* [24] that has been widely explored chemically since the 90s [25,26,27,28].

### 2.2. P3, a High Molecular Weight Natural Product Isolated from Crambe tailliezi Sponge, Selectively Inhibits Protein Kinases Aurora A and Aurora B

As the dichloromethane–methanol extract E13 from *Crambe tailliezi* was shown to inhibit few protein kinases from the panel tested, this crude organic extract was selected to isolate the active compound. The marine natural product P3 was thus found as the main component of the extract (see the materials and methods section for details and Figure S1).

**Figure 3:**
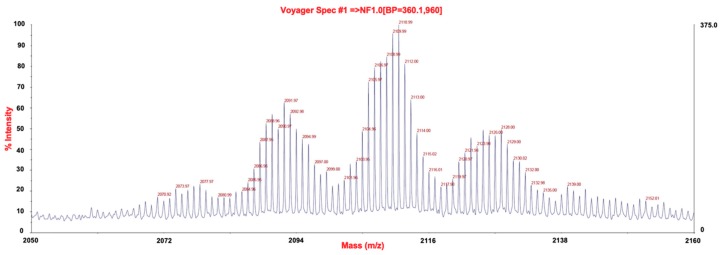
MALDI-TOF analysis of P3 (reflectron positive ion mode, DHB matrix).

**Figure 4:**
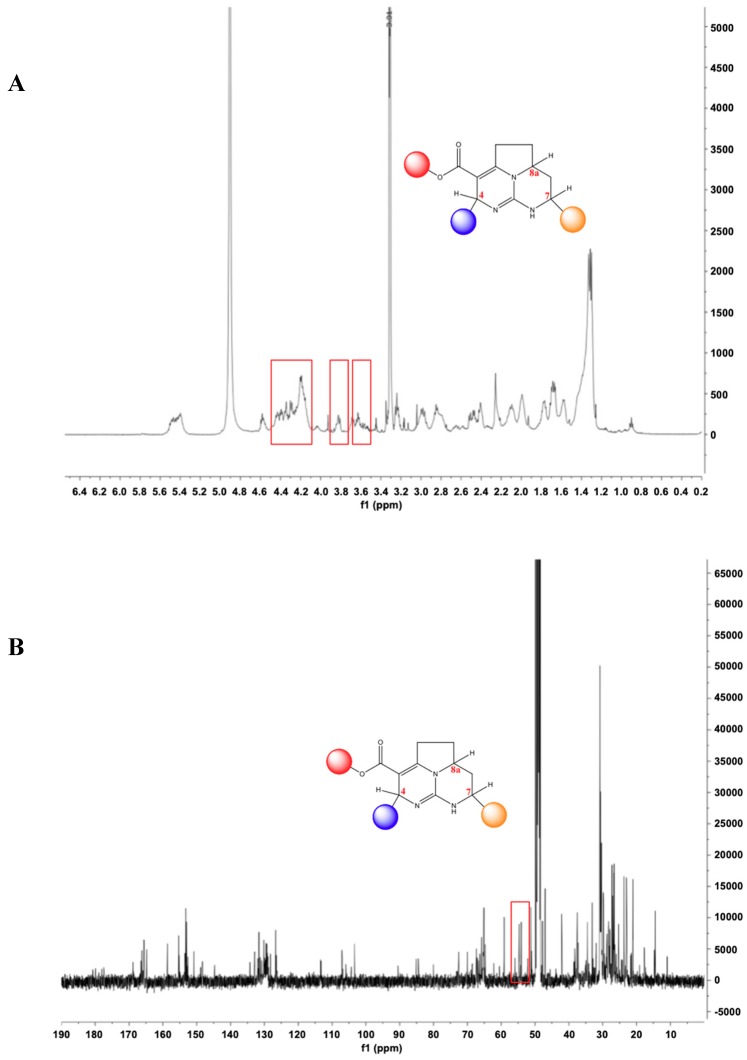
(**A**) ^1^H NMR spectrum of P3 (CD_3_OD, 500 MHz); (**B**) ^13^C NMR spectrum of P3 (CD_3_OD, 125 MHz).

The chemical structure of P3 compound was studied by positive-ion reflectron MALDI-TOF MS analysis (Figure 3) and NMR (Figure 4). Unfortunately, we were unable at this point to determine the chemical structure of P3 compound. The complexity of its structure is highlighted by its estimated molecular size (2200–2800 Da) as shown on Figure 3.

NMR spectra of P3 contain several signals suggestive of a batzelladine-like metabolite [28] (Figure 4). In particular, signals at *δ*_H_ 4.6–4.1 ppm (H_-_6), 3.8 ppm (H8a), 3.6 ppm (H7) in the ^1^H NMR spectrum (Figure 4A) and the signal at δ_c_ 52.0 (C7) in the ^13^C NMR spectrum (Figure 4B) are characteristic of a batzelladine C nucleus [29].

The kinase inhibition properties of the purified product were evaluated. As shown in Table 2, P3 inhibited the kinase activity of Aurora B in a dose-dependent manner with a half-maximal inhibitory concentration (IC_50_) of 2.63 µg/mL. The activity of the extracts E13 and purified product were then tested on Aurora A, a closely related Aurora B kinase, also essential to mitosis. Both the extracts E13 and P3 showed an inhibitory activity on Aurora A, with IC_50_ values of 14.7 and 7.58 µg/mL respectively, in a concentration range close to the one obtained against Aurora B. In comparison with the extract E13, the purified compound showed greater inhibitory activity on both kinases Aurora A and Aurora B (Table 2 and Figure S2), which strongly suggests that P3 is the main if not sole molecule that accounts for the inhibitory activity of E13.

### 2.3. P3 Inhibits the Viability of Human Cancer Cell Lines

The kinase-based screening showed an inhibition of Aurora A and B kinases by P3. These proteins are important for faithful transition through mitosis. They are overexpressed in a wide variety of human tumors and are thus considered as pertinent targets for developing new cancer therapies. Known inhibitors of Aurora A and B, such as Alisertib (MLN8237) [30,31], display antiproliferative activity in a wide range of human tumor cell lines [32,33,34]. The effect of P3 on cell viability was thus explored.

P3 was tested on nine human cancer cell lines: Hep G-2 (hepatocellular carcinoma), MCF-7 (breast adenocarcinoma), U-87 (glioblastoma), SH-SY5Y (neuroblastoma), HT-29 (colorectal adenocarcinoma), U-2 OS (osteosarcoma), PANC-1 (pancreatic epithelioid carcinoma), AsPC-1 (pancreatic adenocarcinoma), A3 (acute T cell leukemia). Four noncancerous cell lines were also tested: hTERT RPE-1 (human retinal pigmented epithelial cell line), RC-124 (kidney primary cell line), HEK-293 (embryonic kidney cell line), HT-22 (mouse hippocampal neuronal cell line). As shown in Table 3, a 24 h exposure with P3 induced a dose-dependent reduction of cell survival in all cell lines tested. When used at 50 µg/mL, P3 decreased over 90% of cell viability after 24 h of treatment (Figure S3A). The half-maximal effective concentrations (EC_50_) of P3 on cells ranged from 6.6 to 22.4 µg/mL (Table 3 and Figure S3B).

**Figure 5:**
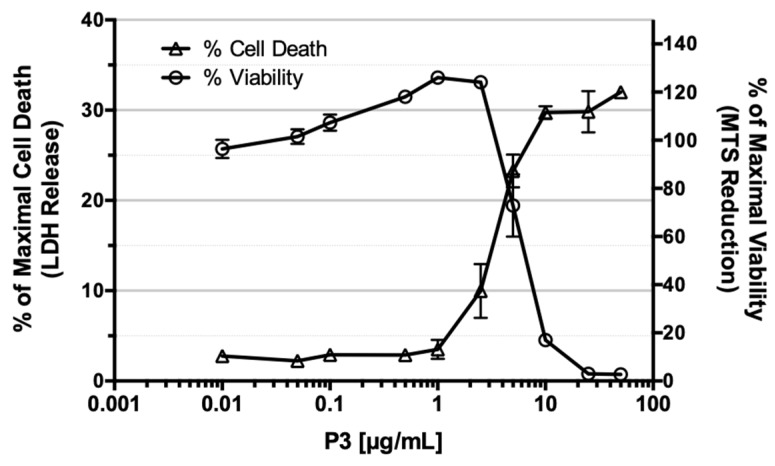
Dose-dependent induction of cell death in U-2 OS cells by P3 product. U-2 OS cells were exposed for 24 h to increasing concentrations of P3 (ranging from 0.01 to 50 µg/mL). Cell viability was assessed by MTS reduction assay and is plotted in % of maximal viability (detected in DMSO-treated cells, right axis). A similar experiment was performed to evaluate cell death (LDH release assay, left axis). Results are plotted in % of maximal LDH release (cell lysis). Data are mean ± SD (*n* = 3).

Further investigations on the mechanism(s) by which P3 affects cell viability were carried out in U-2 OS cells, the most sensitive solid tumor-derived cell line of the panel used here. Cell viability was evaluated by detection of MTS reduction. As the reduction of tetrazolium reagents can be occasionally observed under conditions in which cell death does not occur, the lactate dehydrogenase (LDH) release assay was also used as an independent cell death assay. As shown in Figure 5, the treatment of U-2 OS with increasing doses of P3 product induced a dose-dependent reduction of cell survival that correlated with the similar dose-dependent induction of cell death.

### 2.4. P3 Induces Apoptotic Death in Osteosarcoma U-2 OS Cell Line

**Figure 6:**
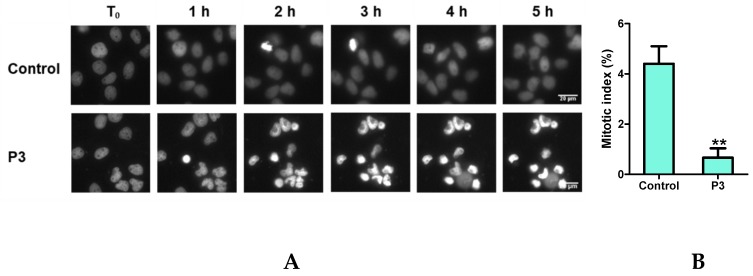

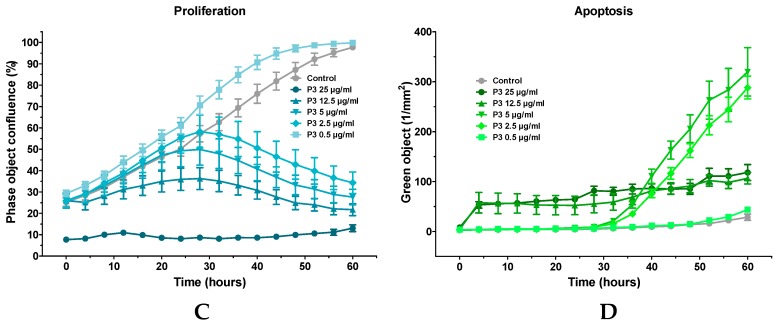
P3 induces apoptosis and affects mitosis in U-2 OS cells. (**A**) Real-time imaging of chromatin dynamics in P3-treated U-2 OS cells expressing Histone H2B-RFP. Image recording started 4 h after treatment with 7.5 µg/mL of P3, by fluorescence microscopy at 20× magnification. (**B**) Mitotic indexes of cells were recorded after 24 h of treatment with 7.5 µg/mL of P3. Five hundred cells/condition were analyzed. Data are mean ± SD (*n* = 3); ** *P* < 0.01, compared with control cells (Student’s *t*-test). Dose- and time-dependent effects of P3 on cell proliferation (**C**) and on apoptosis (**D**) in U-2 OS cells. Cells were stained with annexin V-FITC and treated with P3 (0.5–25 µg/mL) over 60 h. Images were acquired by real-time live-cell imaging. Images were analyzed in order to quantify cell confluence (**C**) and apoptotic cell number (green fluorescent object) (**D**). Data are mean ± SD (*n* = 3).

In order to visualize the dynamics of living cell nuclei, we imaged U-2 OS cells expressing Histone H2B fused to the RFP (red fluorescent protein), treated or not with 7.5 µg/mL of P3. The results obtained indicated that, in P3-treated cells, chromatin became hypercondensed (strong fluorescent signal), starting from 5 h after the beginning of treatment (1 h of movie time) (Figure 6A). By 6 h of treatment, all nuclei showed condensed chromatin, a typical phenotype of apoptotic cells. Note that the cell with condensed chromatin, appearing in the control culture by 2 h of movie time, is in mitosis and ultimately divided (see Supplementary Materials Section, Videos S1 and S2). In addition, the mitotic index (MI), the ratio of mitotic cell number over total cell number, was also measured. A significant decrease of the mitotic index was detected in P3-treated cells (0.7%), compared with control cells (4.4%) (Figure 6B), suggesting that cell division was severely affected.

The dose- and time-dependent effects of P3 on cell proliferation and on externalization of phosphatidylserine (an early apoptosis feature) were then evaluated in U-2 OS cells, by real-time live-imaging (Figure 6C,D). The evolution of cell confluence (Figure 6C) as well as the number of apoptotic cells, by annexin V staining (Figure 6D), were also measured. The results obtained confirmed that treatments with 2.5 to 12.5 µg/mL of P3 inhibited cellular proliferation and induced apoptosis simultaneously. Higher dose of P3 (25 µg/mL) induced massive cell death (Figure 6C,D). To confirm the effect of P3 treatment on tumor cell proliferation, a scratch wound migration assay was used (Figure S4). We observed a delay in wound closure after 48 h of treatment with P3 at 5 and 12.5 µg/mL, which occurred concomitantly to cell death.

### 2.5. Characterization of P3-Induced Apoptotic Death in Osteosarcoma U-2 OS Cells

**Figure 7:**
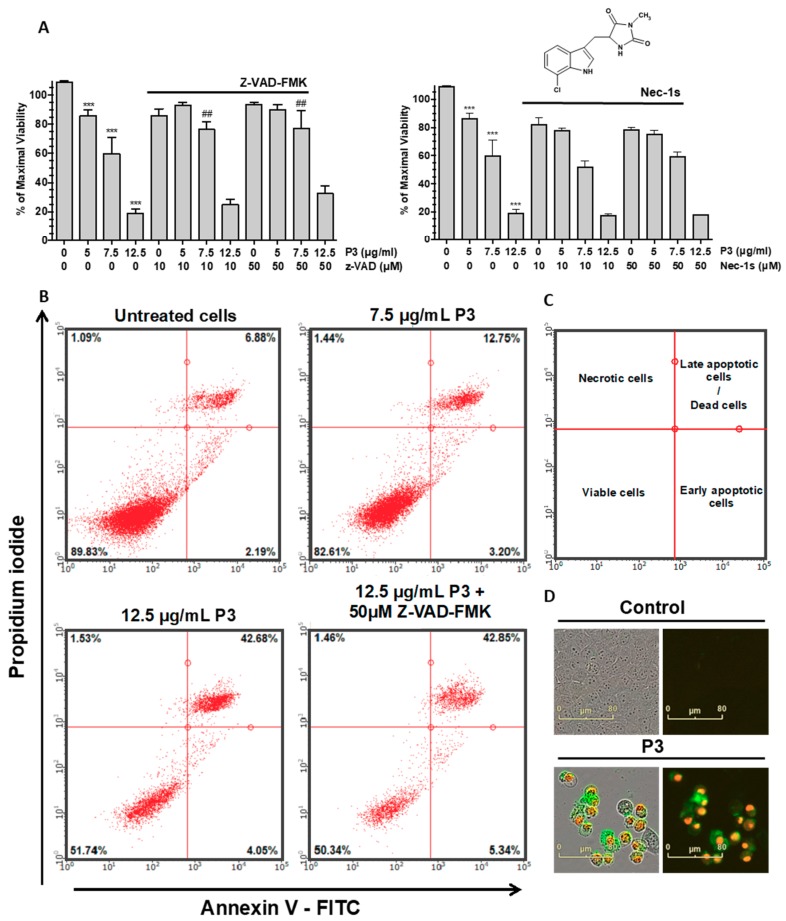
Analysis of apoptosis pathway induced by P3 in U-2 OS cells. (**A**) P3-induced inhibition of cell survival in U-2 OS cells. Cells were treated with Z-VAD-FMK (10 or 50 µM) or Nec-1 (10 or 50 µM) and P3 (7.5 or 12.5 µg/mL) for 24 h. Cell viability was measured by MTS reduction assay. Data are mean ± SD (*n* = 3); *** *P* < 0.001 compared with control cells; ## *P* < 0.01, compared with 7.5 µg/mL P3 without Z-VAD-FMK group (Student’s *t*-test). (**B**) Apoptosis detection by flow cytometry (FACS) analysis after annexin V-FITC/PI staining after a 24 h treatment of U-2 OS cells by DMSO (untreated control cells), 7.5 µg/mL or 12.5 µg/mL of P3 compound and 12.5 µg/mL of P3 compound with 50 µM of Z-VAD-FMK. (**C**) Schematic description of cell repartition in each quadrant from the experiment in B. (**D**) Visualization of the cells treated for 24 h with P3 by microscopy. Cell were labelled as in B, with annexin V FITC (green) et PI (red). Representative merged phase-contrast/fluorescence and fluorescence images were taken at 20× magnification.

We next investigated the type of P3-induced cell death in U-2 OS cells. Cells were treated with three concentrations (5, 7.5, and 12.5 µg/mL) of P3 for 24 h in the presence or in absence of reference inhibitors of two programmed cell death subroutines: (i) Z-VAD-FMK (pan-caspase inhibitor that inhibits caspase-dependent apoptosis) [35] or (ii) necrostatin-1s (nec-1s, specific inhibitor of receptor-interacting kinase-1, RIPK1 kinase, which inhibits necroptosis and RIPK1-dependent apoptosis) [36]. Cell viability was measured using MTS reduction assay. As reported on Figure 7A, P3 strongly reduced cell survival (40% at 7.5 µg/mL and 80% at 20 µg/mL). This effect, obtained with 7.5 µg/mL of P3, was significantly reversed by 10 µM and 50 µM of Z-VAD-FMK, indicating a role of caspases in the mechanism of action of P3 compound (see also the Figure S5, left panel). Oppositely, the effect of P3 on cell survival was not inhibited by 50 µM of nec-1s. This data suggests that RIPK1 is not involved in the cellular effects triggered by P3. High doses of a less specific inhibitor of RIPK1, necrostatin-1 (Figure S5, right panel), were also tested. Interestingly, 50 µM and 125 µM of nec-1 were shown to inhibit significantly the effect of P3 on cell survival, suggesting a possible role of unknown off-targets of nec-1 in the death process initiated by P3.

Consequently, apoptotic cells were quantified by flow cytometry. After 24 h of treatment with P3, U-2 OS cells were stained with both annexin V conjugated to fluorescein isothiocyanate (FITC annexin V) and PI (propidium iodide) to detect necrosis and early or late apoptosis in total cell population (as shown on Figure 7C). We found that P3 induced mostly late apoptosis in treated cells (42.6% of total cell population when treated with 12.5 µg/mL of P3, Figure 7B). The effect of 50 µM of Z-VAD-FMK was also tested and showed that this high dose of pan-caspase inhibitor cannot inhibit the late apoptosis triggered by P3.

The P3-induced apoptosis was also observed by microscopy (Figure 7D). It is accompanied with apoptosis morphological changes (cell roundup and nuclear condensation) and with positive signals for both annexin V and PI staining, the hallmark of the late apoptosis phase.

### 2.6. P3 Affects U-2 OS Spheroids Viability and Integrity

**Figure 8:**
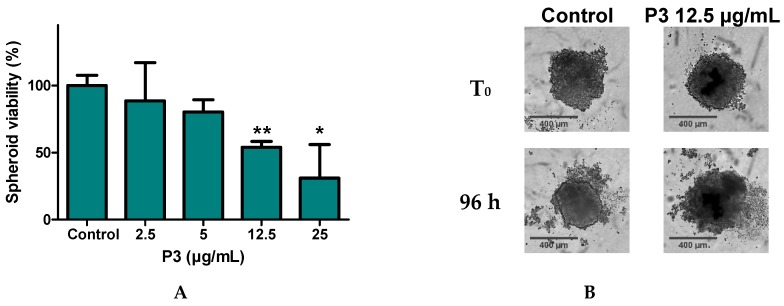
Effects of P3 on the growth and viability of U-2 OS spheroids. (**A**) Dose-dependent effect of a 96 h treatment with P3 (from 2.5 to 25 µg/mL) on the viability of U-2 OS spheroids. Spheroid viability was quantified by MTS reduction assay. Data are mean ± SD (*n* = 3); * *P* < 0.05, ** *P* < 0.01, compared with DMSO-treated control spheroids (Student’s *t*-test). (**B**) Spheroid morphology was analyzed by microscopy. Bright field images showing the morphology of U-2 OS spheroids were taken at 4× magnification using IncuCyte^®^ (Sartorius, Ann Arbor, MI, USA.).

As the effect of P3 on programmed cell-death of two-dimensional (2D) monolayer cells was validated, we next investigated in vitro effect on single spheroids as experimental models of three-dimensional (3D) cell populations. Multicellular spheroid cultures were initially developed in 1970 to recapitulate the functional phenotype of human tumor cells and their responses to radiotherapy [37,38]. Compared to the traditional 2D format, the 3D spheroid system provides a more accurate and reproducible model for drug development: they are able to closely mimic the main features of human solid tumors, including the structural organization, increased cell survival as well as nutrient, metabolite, and oxygen gradients. Numerous studies have shown a good correlation between 3D- culture experiments and clinical trials, especially in human solid tumors [39]. In order to study the effect of P3 on 3D-cell culture, U-2 OS spheroids were generated and treated them with P3. The viability of spheroids was estimated by MTS reduction assay after a 96 h treatment. As shown in 6A, treatments with 12.5 and 25 µg/mL of P3 significantly reduced the viability of spheroids. To complete this analysis, the morphology as well as the size of spheroids were next observed by microscopy. The data obtained indicated that a treatment of U-2 OS spheroids with 12.5 µg/mL of P3 expanded and collapsed the three-dimensional cellular organization of the spheroids (Figure 8B).

## 3. Discussion

In the present work, we used a protein kinase target-based screening strategy to identify extracts from marine organisms with potential bioactivity. We isolated a high molecular weight metabolite, P3, from the sponge *Crambe tailliezi* (sampled in the Mediterranean Sea) as a new cytotoxic marine natural product that inhibits the Aurora protein kinases. Note here that one of the 50 FDA-approved protein kinase inhibitors is Midostaurin (as Rydapt^®^, Novartis Pharmaceuticals, Inc.). This direct derivative of staurosporine is a multitarget kinase inhibitor for the treatment of adult patients with newly diagnosed acute myeloid leukemia (AML) who harbor mutations in FMS-like tyrosine kinase 3 (FLT3) [40]. Both are indolocarbazole alkaloids that can be found in numerous marine species from invertebrates (e.g., from the ascidian *Eudistoma toealensis*) to marine sponge-associated actinomycetes [15,41].

Within the Crambeidea family, *Crambe tailliezi* [23], is an encrusting marine sponge also scarcely distributed in the Mediterranean and the Macaronesian Seas, which remains poorly investigated for either its chemical composition or the biological properties of its metabolites, contrary to *Crambe crambe* [24] that has been widely explored chemically since the 90s [25,26,27]. Among the compounds already described in *C. crambe*, the guanidine alkaloids, crambescins, and crambescidins showed a wide range of bioactivities such as antibacterial, antiviral, and antifungal properties and cytotoxic activities against several cancer cell lines [28,42,43,44]. The present study is thus focused on an untapped marine resource.

The primary screening performed on a selected panel of protein kinases showed that *C. tailliezi* P3 is a dual inhibitor of Aurora A and B kinases. Aurora kinases are known to play pleiotropic roles during mitosis. They are notably important for the capture, alignment, and segregation of chromosomes and thus were subjects of intensive screening campaign to characterize new potent inhibitors: 50+ clinical trials were performed in solid and hematological tumors [45]. Following the screening of kinases, we showed that P3 decreased the viability of two- and three-dimensional cell cultures and induced apoptosis of U-2 OS cells.

Numerous cell death subroutines are now described and classified as reported by Galluzzi et al. [4]. Here, we showed that P3 induces an apoptotic-like programmed cell death. Indeed, these results suggested that caspases have no crucial role in the process triggered by P3. As shown by FACS analysis, even cotreated with 50 µM of Z-VAD-FMK, the cells died by late apoptosis (PI+/Annexin V-FITC+). It has been proposed that no single experimental system exists in which Z-VAD-FMK can save cells from dying [46]. Consequently, additional studies will be crucial to prove that active caspases are not a prerequisite for execution of cell death induced by P3. Interestingly, the effect of P3 on cell survival was not inhibited by 50 µM of nec-1s, whereas the same dose of a less specific inhibitor of RIPK1, nec-1, can inhibit this phenotype. This indicates that P3 induces necrostatin-1-inhibitable cell death independent of RIPK1 kinase as it was previously observed for nitric oxide-induced cell-death of pancreatic β-cells [47]. It would be now very important to characterize the targets of necrostatin-1 that are responsible for this inhibition. This chemobiological approach should provide crucial insights to pinpoint the molecular pathway involved in P3-induced cell death.

Structural determination of P3, based on chemical degradation, NMR, and MS analyses, was unsuccessful due to the low quantities of P3 and its associated fragments recovered after isolation. Similarly, another preliminary study reported also that isolation and structure elucidation of the major compound produced by *C. tailliezi* is extremely complicated [48]. New strategies have now to be explored such as the cocrystallization of purified P3 metabolite with Aurora A or B kinase following X-ray structure determination.

## 4. Materials and Methods

### 4.1. Sample Material

*Cliona viridis* (Schmidt, 1862), *Axinella polypoides* (Schmidt, 1862), *Axinella* sp., *Cacospongia* sp., *Crambe crambe* (Schmidt, 1862), *Crambe tailliezi* (Vacelet & Boury-Esnault, 1982), *Acanthella acuta*, *Agelas oroides* (Schmidt, 1864), *Hemimycale columella* (Bowerbank, 1874), *Ircinia oros* (Schmidt, 1864), *Ircinia variabilis* (Schmidt, 1862), *Phorbas topsenti* (Vacelet & Pérez, 2008), *Pleraplysilla spinifera* (Schulze, 1879), *Pseudaxinyssa* sp., *Reniera fulva* (Topsent, 1893), *Reniera mucosa* (Griessinger, 1971), *Reniera sarai* (Pulitzer-Finali, 1969), *Sarcotragus foetidus* (Schmidt, 1862), *Sarcotragus spinosulus* (Schmidt, 1862), were sampled in Villefranche-sur-Mer (France) in 2011. *Aplidium* sp., *Cystodytes* sp., *Halocynthia papillosa* (Linnaeus, 1767), *Polysyncraton* sp., *Haliclona mediterranea* (Griessinger, 1971), *Hexadella* sp., *Oscarella* sp. *Axinyssa* sp. were sampled in Pota del Llop (Spain) in 2012. All the marine species were identified by Philippe Amade and Mathieu Foulquié.

### 4.2. Preparation of Natural Extracts and of P3 Pure Compound

All the solvents (analytical grade), formic acid and 2,5-dihydroxybenzoic acid (DHB) were purchased from Sigma Aldrich (France). NMR (^1^H and ^13^C) and MALDI-TOF analyses were performed as described in [49].

HPLC-PDA-ELSD analyses were performed with a Waters Alliance 2695 HPLC system (Waters Corporation, Milford, MA) coupled with a Waters 996 photodiode array detector and a Sedex 55 evaporative light-scattering detector (SEDERE, France), using a bifunctional Macherey-Nagel NUCLEODUR^®^ Sphinx RP column (250 × 4.6 mm, 5 μm) consisting of a balanced ratio of propylphenyl and C18 ligands. The mobile phase was composed of H_2_O (plus 0.1% HCO_2_H) and acetonitrile (CH_3_CN plus 0.1% HCO_2_H) and the following gradient was used: H_2_O:CH_3_CN 90:10 for 5 min, H_2_O:CH_3_CN 90:10 to 0:100 for 30 min, 0:100 for 5 min, 0:100 to 90:10 for 15 min (flow: 1.0 mL.min^-1^, injection volume: 20 µL). Chromatograms were extracted at the following detection wavelengths for visual inspection: 214, 254, and 280 nm.

Each freeze-dried species was grounded to give a dry powder (1 g), extracted three times during 10 min with 10 mL of a mixture of MeOH/CH_2_Cl_2_ (1:1, v/v) in an ultrasonic bath. The filtrates of each extraction were combined, mixed with 0.5 g of RP C18 silica gel and evaporated to dryness. The latter were then loaded onto C18 solid phase extraction (SPE) cartridges beforehand conditioned (2 g, Phenomenex Strata). The SPE columns were first washed with 10 mL of H_2_O for desalting and then eluted with 10 mL of MeOH/CH_2_Cl_2_ (1:1, v/v) in a 20 mL volumetric flask. The resulting organic phase, obtained after concentration under reduced pressure, were used for HPLC–PDA–ELSD analyses and biological screenings.

Desalted crude organic extract of *Crambe tailliezi* (128 mg) was fractionated by size exclusion chromatography (Sephadex LH-20) in MeOH to afford six fractions (Fr. 1–6). Fraction 3 yielded pure compound P3 (46.8 mg).

Finally, each crude organic extract was analyzed by HPLC/ESI-MS/MS in both positive and negative ion modes (see [50] for details on the methods used).

### 4.3. Protein Kinase Assays

In order to measure kinase activity, the ADP-Glo^TM^ assay kit (Promega, Madison, WI, USA) was used according to manufacturer’s recommendations (see [51] for details on this method). The experimental conditions used to perform the various kinase assays are reported in Supplementary Table S1.

### 4.4. Cell Lines and Cell Culture

Dulbecco’s Modified Eagle’s Medium (DMEM), RPMI 1640 Medium, DMEM/F-12 Medium, Fetal Bovine Serum (FBS), Penicillin-Streptomycin (5000 U/mL), Trypsin-EDTA (0.25%), Phosphate-Buffered Saline (PBS) were obtained from Thermo Fisher Scientific (USA). The human cell lines A3, U-2 OS, SH-SY5Y, U-87, Hep G-2, MCF-7, AsPC-1, HT-29, PANC-1, hTERT RPE-1, HEK-293, RC-124, and the mouse cell line HT-22 used in this study were obtained from ATCC (American Type Culture Collection, Rockville, MD, USA) and cultured in medium supplemented with 10% FBS, 50 U/mL penicillin and 50 µg/mL streptomycin. The cells were incubated under a 5% CO_2_ atmosphere at 37 °C and passed twice a week for a use within two months.

### 4.5. Cell Viability and LDH-Release Assays

Evaluation of cell viability: From 5.0 × 10^4^ to 2.0 × 10^5^/mL cells were seeded in 96-well plates (#CC7682-7596, CytoOne®, USA) following 24 h of incubation. Cells were exposed to compounds for 24 h, 48 h, or 72 h. Cell viability was determined by MTS reduction assay (CellTiter 96® AQueous One Solution Cell Proliferation Assay from Promega, Madison, WI, USA), according to the manufacturer’s instructions. This method is based on the reduction of MTS tetrazolium compound by viable cells to generate a colored formazan product. After treatment with the tested compounds, cells were incubated with MTS (3-(4,5-dimethylthiazol-2-yl)-5-(3-carboxymethoxyphenyl)-2-(4-sulfophenyl)-2H-tetrazolium, Inner salt) for 3 h at 37 °C. The absorbance was measured with microplate reader BioTek EL800 (BioTek Instruments, Inc., USA) at 490 and 630 nm. The percentage of viability was calculated by comparing values obtained from treatments with tested compounds to those of DMSO (used as control). Staurosporine, Z-VAD-FMK and necrostatin-1 were purchased from Enzo Life Science (Villeurbanne, France).

Evaluation of cell death: 5.0 × 10^3^ U-2 OS-cells were seeded per well in 96-well plates following 24 h of incubation. Cells were exposed to P3 natural product for 24 h. Cell death was evaluated by measurement of activity of lactate dehydrogenase (LDH). LDH is a stable enzyme normally found in the cytosol of all cells but rapidly releases into the supernatant upon damage of plasma membrane. The % of maximal cell death was determined by using the CyQUANT™ LDH Cytotoxicity Assay Kit (Invitrogen, Carlsbad, CA, USA), according to the manufacturer’s instructions.

### 4.6. Time-Lapse Assay

The cell line U-2 OS H2B-RFP, which expresses Histone 2B fused to red fluorescent protein, was used to observe chromosome dynamics in live cells. Cells (25 000 cells/well) were seeded in four-well plates adapted to video-microscopy (Lab-Tek II Chamber coverglass, Thermo Fisher Scientific, USA) for 24 h. Cells were exposed to P3 or DMSO for 24 h. During the treatment time, cells were visualized under an inverted fluorescence microscopy (Zeiss, Germany) and images were recorded every 20 min by an EMCCD camera (Zeiss, Germany). Images were analyzed by MetaMorph^®^ Software (MetaMorph Inc., USA).

### 4.7. Live-Cell Imaging of Apoptosis in U-2 OS

U-2 OS cells (5 000 cells/well) were seeded in 96-well plates (#CC7682-7596, CytoOne®, USA) 24 h prior to treatment. Cells were then exposed to compounds for as long as 60 h. During the treatment, cell morphology was observed and images recorded using an automatic microscope IncuCyte^®^ S3 Live-Cell Analysis System (Sartorius, Ann Arbor, MI, USA). Moreover, cells were stained with FITC Annexin V Apoptosis Detection Kit I (#556547, BD Biosciences, USA) to detect cell apoptosis. Cell confluence (phase object) and apoptotic cell number (green fluorescent object) were analyzed with IncuCyte^®^ S3 Software.

### 4.8. Detection of Apoptosis by Flow Cytometry

In early apoptotic cells, phosphatidylserine (PS) is translocated from the inner to the outer leaflet of the plasma membrane, thus exposing PS to the external cellular environment. Annexin V labelled with fluorescein has a high affinity for PS exposed on the outer leaflet and is used to identify apoptotic cells [52,53]. PI intercalating DNA enters the cells in the case of late apoptosis or necrosis where the cellular membrane becomes permeable [54]. Living cells give negative signals with both annexin V and PI. In this assay, U-2 OS cells (50 000 cells/well) were seeded in 12-well plates (#CC7682-7512, CytoOne®, USA) for 24 h. Cells were exposed to compounds for 24 h. Cells were harvested, washed twice with cold PBS, and then resuspended in 1× Binding Buffer. Finally, cells were stained with FITC Annexin V Apoptosis Detection Kit I (#556547, BD Biosciences, USA) for 15 min at RT (25 °C) in the dark. The stained cells were analyzed by flow cytometer BD FACSCanto II (BD Biosciences, USA). Data were analyzed with BD FACSDiva Software 8.0 (BD Biosciences, USA). Each experiment was performed in duplicate.

### 4.9. Scratch Wound Assay to Evaluate the Cellular Migration and Proliferation

U-2 OS cells (20 000 cells/well) were grown in DMEM with 10% FBS in 96-well ImageLock plates (#4379, Essen BioScience, USA) for 6 h to reach approximately 100% of confluence. The ImageLock^®^ plate technology is enabled by fiducial markers on the bottom of the plate which provide points from which image locations can be accurately referenced. After creating wounds using the IncuCyte^®^ WoundMaker (96-pin wound making tool), cells were washed twice with DMEM then exposed to compounds for 72 h. During the treatment, cell morphology was observed by using IncuCyte^®^ S3 Live-Cell Analysis System (Sartorius, Ann Arbor, MI, USA). Data were analyzed with IncuCyte^®^ S3 Software. Data were analyzed with IncuCyte^®^ S3 Software.

### 4.10. Spheroid Assay

U-2 OS cells (5000 cells/well) were seeded in Corning® 96-well Round Bottom Ultra Low Adhesion-ULA plates (#7007, Corning Incorporated, USA). After being centrifuged at 1000 RPM for 10 min, plates were incubated under a 5% of CO_2_ at 37 °C for 72 h to produce spheroids (400 µm diameter). Spheroids were then treated with compounds for 96 h. During the treatment, cell morphology was observed using IncuCyte^®^ S3 Live-Cell Analysis System (Sartorius, Ann Arbor, MI, USA). Data were analyzed with IncuCyte^®^ S3 Software: the size of the spheroids was measured using an automated algorithm that masked the largest brightfield object in the field of view. After 96 h of treatment, viability of spheroids was also quantified by MTS reduction assay (CellTiter 96® AQueous One Solution Cell Proliferation Assay from Promega, Madison, WI, USA).

### 4.11. Statistical Analyses

Data were acquired from a minimum of three independent experiments. They are expressed as means ± SD. Statistical analyses were performed with Student’s *t*-test or one-way ANOVA. All statistical analyses were performed with GraphPad Prism6 software (GraphPad Software, San Diego, CA, USA).

## 5. Conclusions

In summary, the results reported here show that the high molecular weight metabolite P3 extracted from the Mediterranean sponge *C. tailliezi* is able to inhibit Aurora A and B protein kinases and to induce apoptotic regulated cell death in U-2 OS human osteosarcoma cells. Taken together, this study should motivate follow up studies on the metabolites from *C. tailliezi* as a new putative source of anticancer compounds. The purpose of this work was also to underline the use of kinase-based screening as a potent approach to identify new bioactive products from untapped marine resources. Protein kinases constitute one of the largest protein families, accounting for approximately 2% of the genes in any given eukaryotic genome, and they are critically involved in the regulation of all cellular processes and signaling pathways [55]. The workflow described in this study can be applied to the vast majority of marine extracts and to a wide spectrum of therapeutic applications.

## Figures and Tables

**Table 1:** Target-based screening of extracts from various marine organisms from Mediterranean Sea. Characterization of extracts with kinase inhibitory activity.

Extract	Marine organism	Hs_CDK2/CyclinA	Hs_CDK5/p25	Hs_CDK9/CyclinT	Hs_Haspin	Hs_PIM1	Rn_DYRK1A	Hs_CK1ε	Hs_GSK3α	Hs_GSK3β	Hs_RIPK3	Hs_AuroraB
**E1**	Ascidium *Aplidium* sp.	81	≥100	100	63	≥100	97	94	≥100	76	87	99
**E2**	Ascidium *Cystodytes* sp.	68	58	52	25	62	29	89	54	70	77	83
**E3**	Ascidium *Halocynthia papillosa*	85	73	81	65	90	95	≥100	91	97	91	100
**E4**	Ascidium *Polysyncraton* sp.	69	82	≥100	94	92	≥100	≥100	≥100	88	86	90
**E5**	Sponge *Acanthella acuta*	100	≥100	83	86	≥100	87	78	≥100	69	≥100	≥100
**E6**	Sponge *Agelas oroides*	71	74	46	26	67	19	46	23	35	80	69
**E7**	Sponge *Axinella polypoides*	≥100	≥100	87	84	≥100	81	77	≥100	67	≥100	≥100
**E8**	Sponge *Axinella* sp.	98	≥100	91	93	87	63	82	68	71	87	≥100
**E9**	Sponge *Axinyssa* sp.	71	63	80	96	≥100	51	≥100	96	88	96	83
**E10**	Sponge *Cacospongia* sp.	≥100	≥100	71	79	100	≥100	92	88	68	93	96
**E11**	Sponge *Cliona viridis*	64	≥100	94	≥100	≥100	85	98	100	85	95	100
**E12**	Sponge *Crambe crambe*	66	62	47	≥100	16	37	19	84	≥100	≥100	79
**E13**	Sponge *Crambe tailliezi*	77	57	48	78	54	27	22	21	28	87	8
**E14**	Sponge *Haliclona mediterranea*	91	≥100	89	87	≥100	61	96	84	≥100	97	100
**E15**	Sponge *Hemimycale columella*	≥100	≥100	≥100	84	≥100	100	≥100	92	62	91	94
**E16**	Sponge *Hexadella* sp.	98	65	7	17	63	4	2	2	10	65	61
**E17**	Sponge *Ircinia oros*	40	≥100	30	34	≥100	26	17	13	9	72	91
**E18**	Sponge *Ircinia variabilis*	100	92	58	42	75	50	≥100	41	37	88	94
**E19**	Sponge *Oscarella* sp.	66	≥100	96	89	92	≥100	≥100	≥100	≥100	97	98
**E20**	Sponge *Phorbas topsenti*	≥100	≥100	91	77	≥100	75	88	93	69	98	≥100
**E21**	Sponge *Pleraplesila spinifera*	≥100	≥100	78	79	≥100	≥100	≥100	98	54	90	87
**E22**	Sponge *Pseudaxinyssa* sp.	96	≥100	31	12	≥100	35	1	5	25	84	≥100
**E23**	Sponge *Reniera fulva*	65	≥100	≥100	61	≥100	83	≥100	100	58	≥100	99
**E24**	Sponge *Reniera mucosa*	88	86	28	25	96	50	17	14	45	64	43
**E25**	Sponge *Reniera sarai*	≥100	76	36	11	50	42	42	28	51	86	86
**E26**	Sponge *Sarcotragus foetidus*	60	≥100	66	14	79	29	21	13	4	92	97
**E27**	Sponge *Sarcotragus spinosulus*	65	≥100	62	35	40	6	7	8	12	86	91

Kinase activities in the presence of extracts (50 µg/mL) were measured by ADP-Glo luminescent assay (Promega, Madison, WI, USA), using 10 µM ATP. Data are mean (*n* = 2) expressed in % of maximal activity, compared with a DMSO control. The red color scale is used to highlight the values that are below 50% of residual kinase activity. CDK: cyclin-dependent kinase, Haspin: haploid germ cell-specific nuclear protein kinase, PIM: proto-oncogene proviral integration site for moloney murine leukemia virus, DYRK1A: dual specificity tyrosine phosphorylation regulated kinase 1A, CK1: casein kinase 1, GSK3: glycogen synthase kinase 3, RIPK: receptor-interacting protein kinase. Kinases are from human origin, *Hs, Homo sapiens*, unless specified *Rn, Rattus norvegicus*.

**Table 2:** IC_50_ values obtained for P3 natural product from the kinase inhibition assay.

Extract/Compound	Marine organism	IC_50_ (µg/mL)	
		Aurora A	Aurora B
E12	Sponge *Crambe crambe*	N/A	124.9
E13	Sponge *Crambe tailliezi*	14.7	7.51
P3	Sponge *Crambe tailliezi*	7.58	2.63

Extracts and P3 compound were tested at various concentrations on human Aurora A and B kinases to calculate the IC_50_ values. Activities were assessed in duplicate using the ADP-Glo luminescent assay, using 10 µM ATP. N/A: nonactive at the maximal concentration tested.

**Table 3:** Effect of P3 on the viability of various cell lines.

Cell type	Cell line	Tissue	EC_50_ (µg/mL) ± SD
Leukemia	A3	T lymphocyte	7.0 ± 0.6
Solid malignancies	U-2 OS	Bone	6.6 ± 0.2 *
	SH-SY5Y	Bone marrow	8.9 ± 0.2
	U-87	Brain	10.6 ± 0.5
	Hep G-2	Liver	11.8 ± 1.6
	MCF-7	Breast	12.2 ± 1.6
	AsPC-1	Pancreas	12.4 ± 0.6
	HT-29	Colon	20.4 ± 0.7
	PANC-1	Pancreas	22.4 ± 1.4
Non-malignant	hTERT RPE-1	Retina, eye	7.2 ± 0.4
	HEK-293	Kidney	11.2 ± 0.6
	RC-124	Kidney	13.3 ± 0.8
	HT-22	Brain (mouse)	14.0 ± 1.2

Cells were incubated with increasing doses of P3 (ranging from 0.01 to 50 µg/mL). Human cells were used, except HT-22, an immortalized mouse hippocampal cell line. Cell viability was measured by MTS reduction assay as mentioned in the Materials and Methods. EC_50_ values are determined from the dose–response curves using GraphPad PRISM software (data are mean, *n* = 3; and * *n* = 6 for U-2OS).

## Supplementary Materials

The following are available online at https://www.mdpi.com/1660-3397/17/10/569/s1, Video S1: Time-lapse imaging in living human osteosarcoma cells treated with control (DMSO), Video S2: Time-lapse imaging in living human osteosarcoma cells treated with P3. Figure S1: HPLC-PDA-ELSD chromatogram of P3. Figure S2: Dose-dependent effect of extracts E12, E13 and the compound P3 on Aurora A/B kinases activity. Figure S3: Effect of P3 on cell viability of human cancerous and non-cancerous cells. Figure S4: P3 affects proliferation of U-2 OS cells. Figure S5: Effect of co-treatment with P3 and high concentrations of Z-VAD-FMK (A) or necrostatin-1 (B) on the cell viability of U-2 OS cells. Table S1: Experimental conditions used for protein kinase assays.
