# Supplementary material for: Kinase-Based Screening of Marine Natural Extracts Leads to the Identification of a Cytotoxic High Molecular Weight Metabolite from the Mediterranean Sponge Crambe tailliezi
Source: Mar Drugs. 2019 Oct 9;17(10):569. doi: 10.3390/md17100569 (PMC6836018; doi:10.3390/md17100569)
Supplement: Supplementary file 1 [file marinedrugs-17-00569-s001.zip › marinedrugs-600490 suppl proofed/Nguyen_et_al_Supplementary_Files.pdf]

SUPPLEMENTARY MATERIALS

Kinase-Based Screening of Marine Natural Extracts Leads to the Identification of a Cytotoxic High Molecular Weight Metabolite from the Mediterranean Sponge *Crambe tailliezi*

Thi-Ngoc-Dung Nguyen [1,2], Omid Feizbakhsh [1], Estelle Sfecci [3], Blandine Baratte [1,4], Claire Delehouzé [1,5], Adrien Garcia [3], Corentin Moulin [3], Pierre Colas [1], Sandrine Ruchaud [1,*], Mohamed Mehiri [3,*] and Stéphane Bach [1,4,*]

[1] Integrative Biology of Marine Models Laboratory (LBI2M), Sorbonne Université, CNRS, UMR 8227, Station Biologique de Roscoff, CS 90074, 29688 Roscoff Cedex, France; dnguyenthingoc@sb-roscoff.fr (T.-N.-D. N.); omid.feizbakhsh@sb-roscoff.fr (O.F.); blandine.baratte@sb-roscoff.fr (B.B.); colas@sb-roscoff.fr (P.C.)

[2] Department of Chemical Analysis and Drug Quality Control, Faculty of Pharmacy, University of Medicine and Pharmacy, Ho Chi Minh City, Vietnam

[3] Marine Natural Products Team, Université Côte d'Azur, CNRS, Institut de Chimie de Nice, UMR 7272, 06108 Nice, France; estelle.sfecci@univ-cotedazur.fr (E.S.); adrien.garcia@univ-cotedazur.fr (A.C.); corentin.moulin@univ-cotedazur.fr (C.M)

[4] Sorbonne Université, CNRS, Station Biologique de Roscoff, FR 2424, Plateforme de criblage KISSf (Kinase Inhibitor Specialized Screening facility), Place Georges Teissier, 29682 Roscoff, France

[5] SeaBeLife Biotech, Place Georges Teissier, 29682 Roscoff, France; claire.delehouze@seabelife.com (C.D.)

* Correspondence: sandrine.ruchaud@sb-roscoff.fr (S.R.); mohamed.mehiri@univ-cotedazur.fr (M.M.); bach@sb-roscoff.fr (S.B.); Tel.: +33-298-292-309 (S.R.); +33-492-076-154 (M.M.); +33-298-292-391 (S.B.); Fax: +33-492-076-151 (M.M.); +33-298-292-526 (S.R. and S.B.)

1. Videos

Video S1: Time-lapse imaging in living human osteosarcoma cells treated with control (DMSO) (cf. AVI video file).

Video S2: Time-lapse imaging in living human osteosarcoma cells treated with P3 (cf. AVI video file).

2. Figures

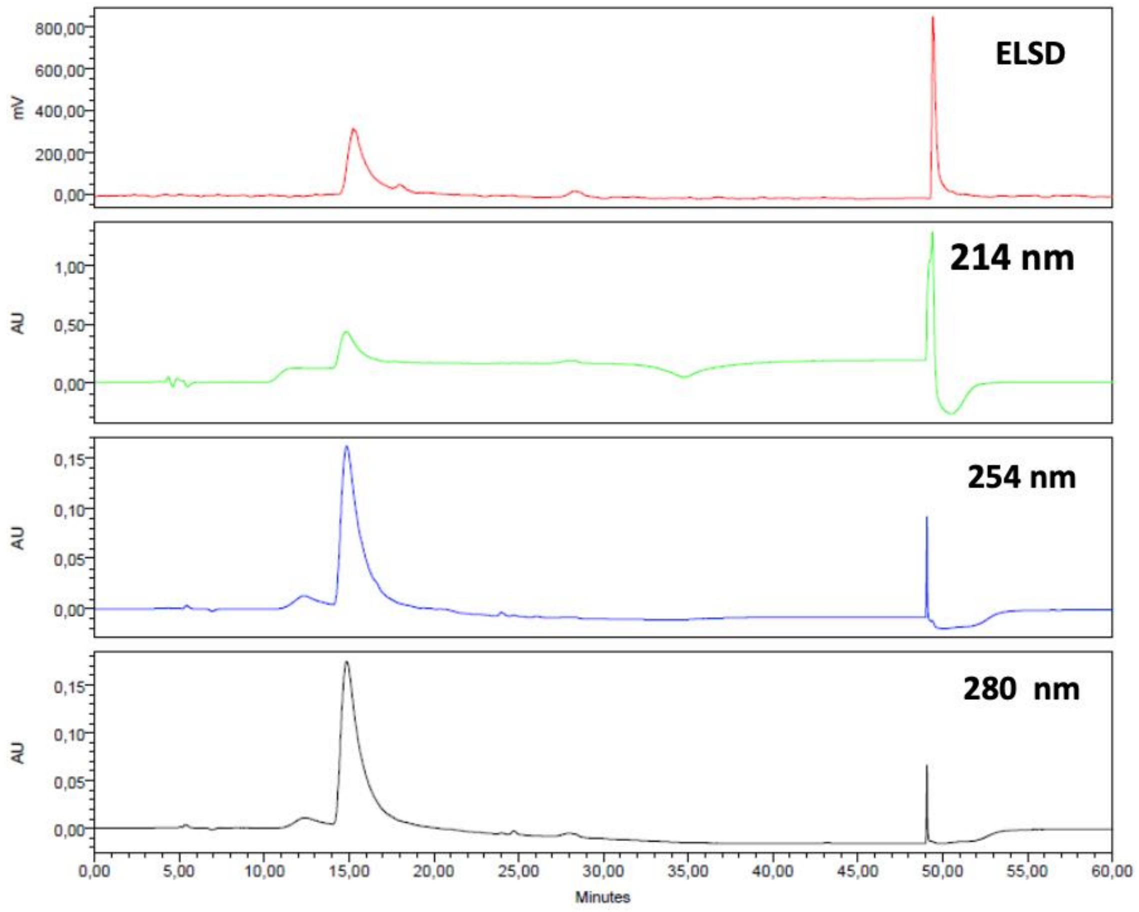

Figure S1. HPLC-PDA-ELSD chromatogram of P3. Bifunctional Macherey-Nagel NUCLEODUR® Sphinx RP column (250 x 4.6 mm, 5 μm) consisting of a balanced ratio of propylphenyl and C18 ligands, gradient H_2O: CH_3CN 90:10 for 5 min, 90:10 to 0:100 for 30 min, 0:100 for 5 min and 0:100 to 90:10 for 15 min (flow: 1.0 mL.min^{-1}, injection volume: 20 μL).

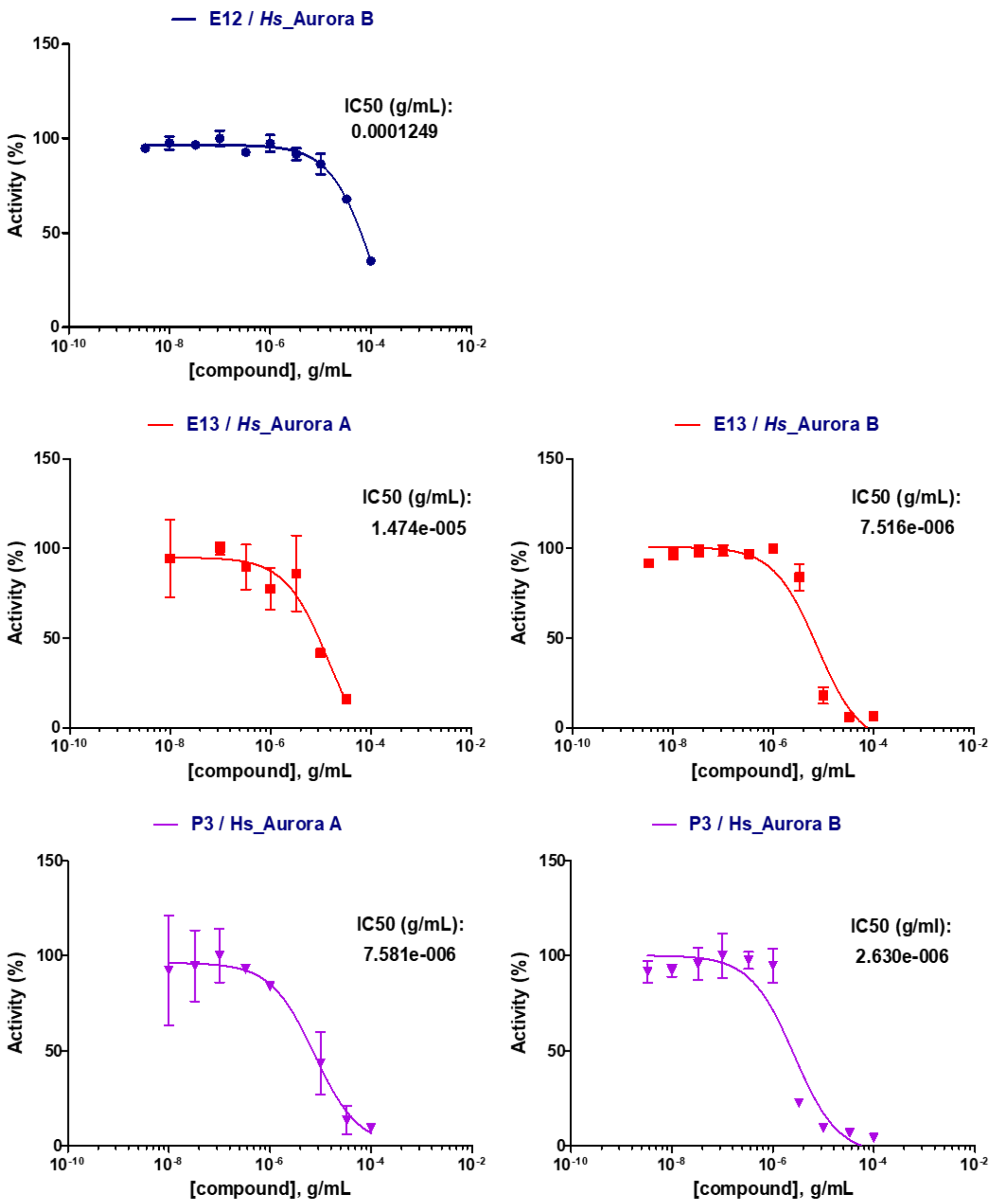

Figure S2. Dose-dependent effect of extracts E12, E13 and of the compound P3 on Aurora A/B kinase activiies. Enzymatic activities were quantified using the ADP-Glo assay in the presence of 10 µM ATP. Data are mean (n=2) expressed in % maximal activity, i.e. measured in the absence of the tested natural product but with an equivalent dose of DMSO (solvent of the marine extracts).

A

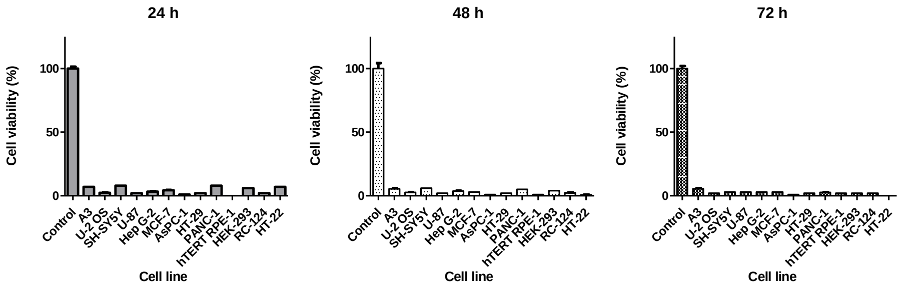

B

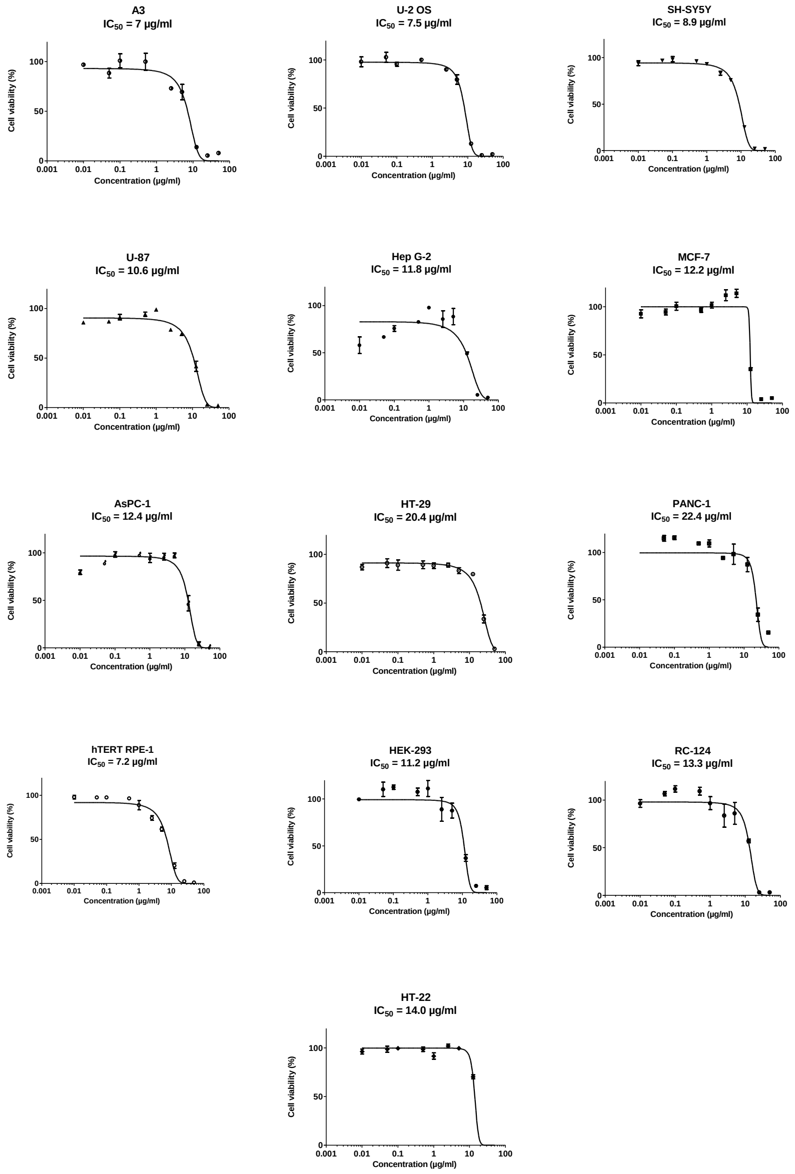

Figure S3. Effect of P3 on the viability of human cancerous and non-cancerous cells: A3, U-2 OS, SH-SY5Y, U-87, Hep G-2, MCF-7, AsPC-1, HT-29, PANC-1, hTERT RPE-1, HEK-293, RC-124 and HT-22 cells. (A) Time-dependent effect of P3. Cells were treated with of P3 (50 μg/mL) for 24 h, 48 h and 72 h. (B) Dose-dependent effect of P3. Cells were treated with increasing concentrations of P3 (0.01-50 μg/mL) for 24 h. Cell viability was measured by MTS assay. Data are mean ± SD (n=3) expressed in % of survival cells, compared with control cells. Half-maximal inhibitory concentrations (IC_{50}) are determined from the curves.

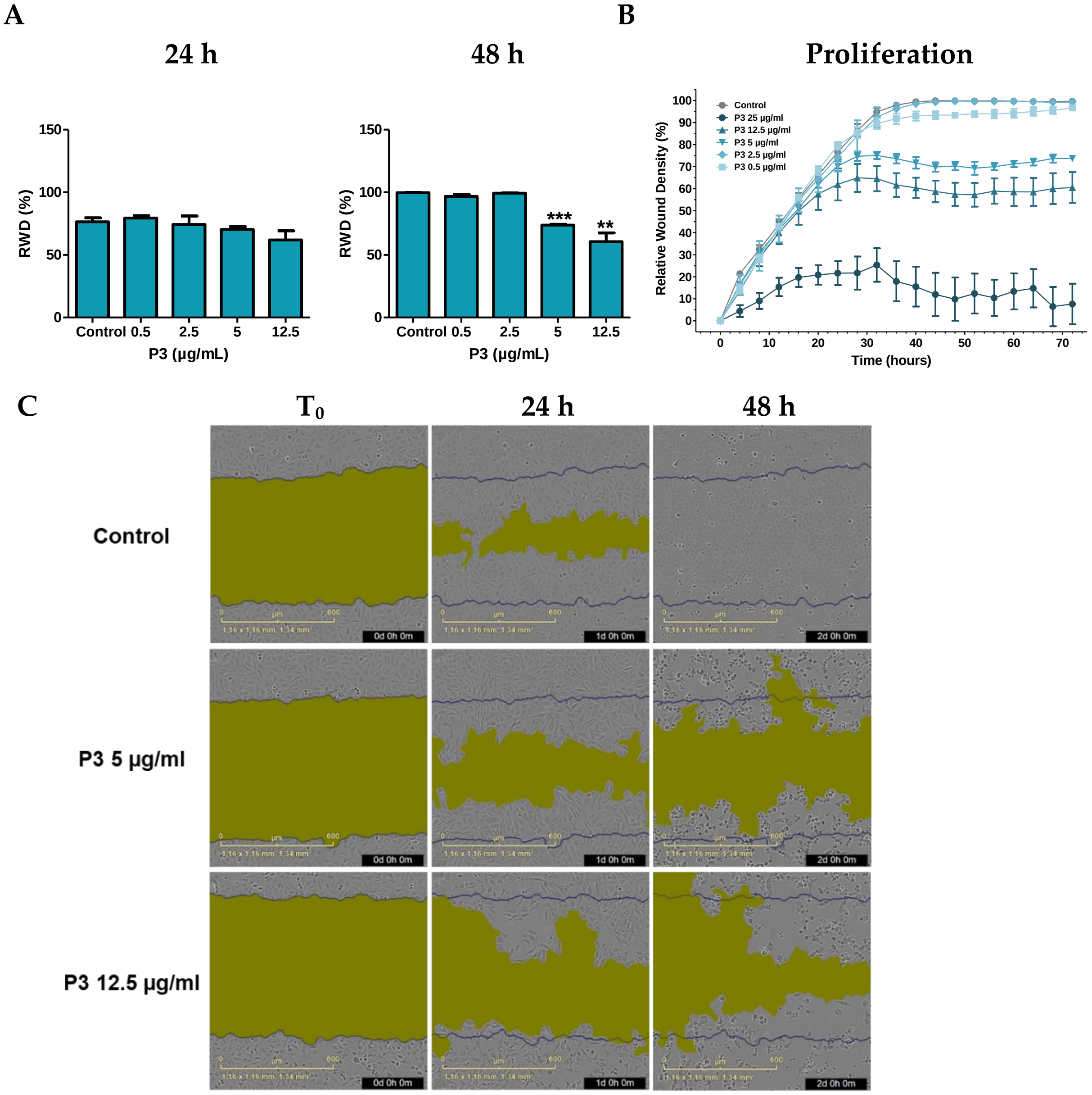

Figure S4. P3 affects proliferation of U-2 OS cells. (**A**) Effect of increasing dose of P3 (0.5 – 12.5 μg/mL) of U-2 OS cells after 24 h and 48 h. (**B**) Dose- and time-dependent effects of P3 on proliferation over 72 h. The Relative Wound Density (RWD) was used as the metrics to estimate the cellular proliferation. Cell-free zones were created by scratch wounds in cell monolayers and the assay was then initiated after addition of P3 or DMSO for the control culture. The progression of cell proliferation was visualized in real time by microscopy. Data are mean ± SD (n=3); ** $P < 0.01$, *** $P < 0.001$ compared with control cells (Student's *t*-test). (**C**) Representative phase-contrast images, taken at 10x magnification, of U-2 OS cells migrating from **A**.

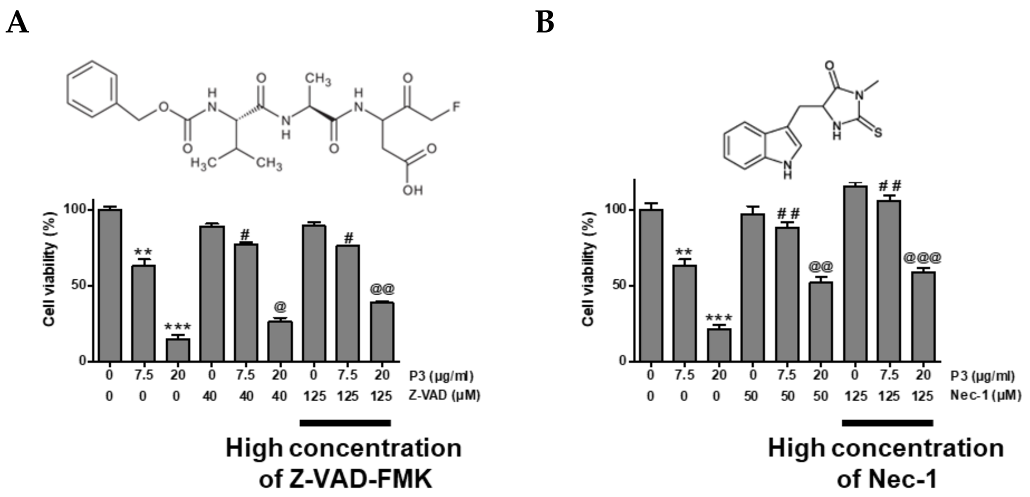

Figure S5. Effect of co-treatment with P3 and high concentrations of Z-VAD-FMK (**A**) or necrostatin-1 (**B**) on the viability of U-2 OS cells. P3-induced inhibition of cell survival in U-2 OS cells. Cells were treated with Z-VAD-FMK (40 or 125 μM) or Nec-1 (50 or 125 μM) for 1 h prior to the treatment with P3 (7.5 or 20 μg/mL) for 24 h. Cell viability was measured by MTS reduction assay. Data are expressed in % of maximal viability (detected in DMSO-treated cells). Data are mean ± SD (n=3); * $P < 0.05$, ** $P < 0.01$, *** $P < 0.001$ compared with control cells; # $P < 0.05$, # # $P < 0.01$, # # # $P < 0.001$ compared with 7.5-μg/mL-P3 treatment group; @ $P < 0.05$, @@ $P < 0.01$, @@@ $P < 0.001$ compared with 20-μg/mL-P3 treatment group (Student's *t*-test).

3. Table

Table S1. Experimental conditions used for protein kinase assays. These enzymatic activities were detected in the presence of 10 μM ATP

Protein Kinase	Enzyme Description	Substrate* *(Working concentration)*	Buffer used**
**Aurora A**	Human, kind gift of Professor Claude Prigent, IGDR Rennes, France	Myelin-Basic Protein, MBP (*0.2 μg/μL*)	**B**
**Aurora B**	Human, recombinant, expressed by baculovirus in Sf9 insect cells, SignalChem product #A31-10G, Richmond, British Columbia, Canada	Myelin-Basic Protein, MBP (*0.2 μg/μL*)	**B**
**CDK2/CyclinA**	Human, kindly provided by Dr. A. Echalier-Glazer, Leicester, UK	Histone H1 (*0.8μg/mL*)	**A**
**CDK5/p25**	Human, recombinant, expressed in bacteria	Histone H1 (*0.8μg/mL*)	**A**
**CDK9/CyclinT**	Human, recombinant, expressed by baculovirus in Sf9 insect cells	Peptide: YSPTSPSYSPTSPSYSPTSPSKKKK (*0.27μg/mL*)	**A**
**CK1ε**	Human, recombinant, expressed by baculovirus in Sf9 insect cells	Peptide: RRKHAAIGSpAYSITA *** (*0.022μg/mL*)	**A**
**HASPIN**	Human, kinase domain, amino acids 470 to 798, recombinant, expressed in bacteria	Histone H3 peptide (1-21): ARTKQTARKSTGGKAPRKQLA (*0.007μg/mL*)	**A**
**PIM1**	Human proto-oncogene, recombinant, expressed in bacteria	Histone H1 (*0.8μg/mL*)	**A**
**RIPK3**	Human, recombinant, expressed by baculovirus in Sf9 insect cells	Myelin-Basic Protein, MBP (*0.1μg/μL*)	**D**
**GSK3α**	Human, recombinant, expressed by baculovirus in Sf9 insect cells	GS-1 peptide: YRRAAVPPSPSLSRHSSPHQSpEDEEE *** (*0.01 μg/μL*)	**A**
**GSK3β**	Human, recombinant, expressed by baculovirus in Sf9 insect cells	GS-1 peptide: YRRAAVPPSPSLSRHSSPHQSpEDEEE *** (*0.01μg/μL*)	**A**
**DYRK1A**	*Rattus norvegicus*, amino acids 1 to 499 including the kinase domain, recombinant, expressed in bacteria, DNA vector kindly provided by Dr. W. Becker, Aachen, Germany	Peptide: KKISGRLSPIMTEQ (*0.033μg/μL*)	**A**

* Peptide substrates were obtained from ProteoGenix (Schiltigheim, France)

** **Composition of the buffers: Buffer A:** 10 mM $MgCl_2$, 1 mM EGTA, 1 mM DTT, 25 mM Tris-HCl pH 7.5, 50 μg/mL heparin; **Buffer B:** 25 mM MOPS, pH 7.2, 12.5 mM β-glycerophosphate, 25 mM $MgCl_2$, 5 mM EGTA, 2 mM EDTA, 0.25 mM DTT; **Buffer D:** 5 mM MOPS pH 7.2, 2.5 mM β-glycerophosphate, 4 mM $MgCl_2$, 2.5 mM $MnCl_2$, 1 mM EGTA, 0.5 mM EDTA, 50 μg/mL BSA, 0.05 mM DTT.

*** "Sp" stands for phosphorylated serine
